# The Synthesis of LiMn*_x_*Fe_1−*x*_PO_4_/C Cathode Material through Solvothermal Jointed with Solid-State Reaction

**DOI:** 10.3390/ma9090766

**Published:** 2016-09-08

**Authors:** Xiangming He, Jixian Wang, Zhongjia Dai, Li Wang, Guangyu Tian

**Affiliations:** 1 Institute of Nuclear & New Energy Technology, Tsinghua University, Beijing 100084, China; hexm@tsinghua.edu.cn (X.H.); wangjixian520@hotmail.com (J.W.); daizj06@126.com (Z.D.); 2 State Key Laboratory of Automotive Safety and Energy, Tsinghua University, Beijing 100084, China; tian_gy@tsinghua.edu.cn; 3 State Key Laboratory of New Ceramic and Fine Processing, Tsinghua University, Beijing 100084, China

**Keywords:** LiMn*_x_*Fe_1−*x*_PO_4_, solvothermal, solid-state reaction, lithium ion batteries

## Abstract

LiMn*_x_*Fe_1−*x*_PO_4_/C material has been synthesized through a facile solid-state reaction under the condition of carbon coating, using solvothermal-prepared LiMnPO_4_ and LiFePO_4_ as precursors and sucrose as a carbon resource. XRD and element distribution analysis reveal completed solid-state reaction of precursors. LiMn*_x_*Fe_1−*x*_PO_4_/C composites inherit the morphology of precursors after heat treatment without obvious agglomeration and size increase. LiMn*_x_*Fe_1−*x*_PO_4_ solid solution forms at low temperature around 350 °C, and Mn^2+^/Fe^2+^ diffuse completely within 1 h at 650 °C. The LiMn*_x_*Fe_1−*x*_PO_4_/C (*x* < 0.8) composite exhibits a high-discharge capacity of over 120 mAh·g^−1^ (500 Wh·kg^−1^) at low C-rates. This paves a way to synthesize the crystal-optimized LiMn*_x_*Fe_1−*x*_PO_4_/C materials for high performance Li-ion batteries.

## 1. Introduction

Thanks to the research of Good enough and co-workers since 1997 [1], olivine LiMPO_4_ (M = Fe, Mn, Co, Ni) have been attracting much attention as cathode materials. Among all the LiMPO_4_ compounds, LiMn*_x_*Fe_1−*x*_PO_4_, retaining high energy density of LiMnPO_4_ as well as stability of LiFePO_4_, is considered as a promising material for its low cost, nontoxicity, and compatibility with commercial electrolytes [2,3]. Previous research suggests that Mn–Fe inter-doping offers LiMn*_x_*Fe_1−*x*_PO_4_ material better rate capability than LiMnPO_4_ and higher energy density than LiFePO_4_ [4]. However, the synthesis of a highly uniform LiMn*_x_*Fe_1−*x*_PO_4_ solid solution is still challenging. Firstly, synthesis by hydrothermal or solvothermal suffers from the segregation of LiMnPO_4_ or LiFePO_4_. Due to different chemical activities among various cations [5,6,7,8], the co-precipitation of Mn^2+^ and Fe^2+^ by a soft chemistry method needs careful control of pH value, concentration, raw material, and solvent, even though the Mn^2+^–Fe^2+^ proportion in the co-precipitation generally falls in a limited range. Secondly, the synthesis through solid-state reaction suffers from poor batch uniformity, which might be caused by the nonuniform cation diffusion or phase separation during the solid reaction process. Thirdly, it is not easy to realize the morphology control for olivine cathode materials, which is very important for improving its electrochemical performances. In previous reports of the synthesis of a LiMn*_x_*Fe_1−*x*_PO_4_ solid solution [9,10], it is not widely and systematically investigated how the LiMn*_x_*Fe_1−*x*_PO_4_ solid solution forms and whether it experiences a mixture of LiMn*_x_*Fe_1−*x*_PO_4_, LiMnPO_4_, LiFePO_4_, or a combination of these introduced by partial phase separation of solid solution. As is known to all, LiMnPO_4_ phase exhibits poor properties ascribed to instability of Mn^3+^ [11,12] and a large volume misfit (11.6%) between lithiated and delithiated phase [13]. With Fe substitution, LiMn*_x_*Fe_1−*x*_PO_4_ solid solution has much better rate capability [14] than LiMnPO_4_ of comparable morphology, which is attributed to reduced volume misfit between coexisting phases and a higher stability of crystal structure [15,16]. It is of great importance to have a clear sight into the phase separation of LiMn*_x_*Fe_1−*x*_PO_4_ solid solution since the electrochemical properties strongly depend on composition. Thus, it is urgent to have a clear and comprehensive investigation on the phase reaction mechanism of LiMn*_x_*Fe_1−*x*_PO_4_ for better application of this type of cathode material.

Considering that LiMn*_x_*Fe_1−*x*_PO_4_ material also needs carbon coating to improve the conductivity before commercial application, we joined solvothermal with solid-state reaction together and made use of a novel access method to synthesize a morphology-regulated LiMn*_x_*Fe_1−*x*_PO_4_/C composite with accurate stoichiometric Mn/Fe composition. Firstly, morphology regulated LiMnPO_4_ and LiFePO_4_ nano-plates were obtained through a solvothermal method. Then, these precursors were carbon-coated with sucrose as a carbon resource. LiMn*_x_*Fe_1−*x*_PO_4_/C composite was obtained through a facile heat treatment during the process of carbon coating. The solid reaction process and phase composition were studied by TG-DSC, XRD and SEM. In addition, the rate properties of LiMn*_x_*Fe_1−*x*_PO_4_/C composites with various value of *x* were compared.

## 2. Experimental Section

### 2.1. Synthesis of LiMnPO_4_ and LiFePO_4_ Nano-Plates

LiMnPO_4_ and LiFePO_4_ nano-plate precursors with length less than 100 nm were synthesized by a solvothermal method seen in our previous work [17,18]. Portions of 0.016 mol MSO_4_ (M = Mn, Fe) and 0.048 mol LiOH·H_2_O were respectively dissolved in 20 mL mixture solvents of ethylene glycol and deionized water (volume ratio 4:1) and then mixed with 0.016 mol H_3_PO_4_ in a particular feeding sequence. Nano-plates were obtained through solvothermal reaction at 180 °C for 12 h. LiFePO_4_ plates with lengths of around 500 nm and LiFePO_4_ micro-spheres with diameters over 5 μm were synthesized by other reported solvothermal methods [19].

### 2.2. Synthesis of LiMn_x_Fe_1−x_PO_4_/C Composite

The as-prepared LiMnPO_4_ and LiFePO_4_ nano-plates in various molar ratios were mixed with 15% of sucrose in weight and milled for 15 min. Then, the mixed powder was calcined in nitrogen flow at 650 °C for 5 h to obtain the LiMn*_x_*Fe_1−*x*_PO_4_/C composite. To investigate the process of solid-state reaction, we calcined the mixture of LiMnPO_4_ and LiFePO_4_ nano-plates at different temperatures for various heating times.

### 2.3. Materials Characterization

X-ray powder diffraction patterns of the composites were characterized on a Bruker D8 Advance X-ray diffractometer (Karlsruhe, Germany) in a Bragg-Brentano configuration with Cu K_α1_ and Cu K_α2_ radiation (λ = 0.15418 nm). The morphology and element distribution of the composites were inspected with a scanning electron microscope (SEM, JSM-5600LV, JEOL, Tokyo, Japan), a transmission electron microscope (TEM, H-800, Hitachi, Tokyo, Japan), a scanning transmission electron microscopy (STEM, H-800, Hitachi, Tokyo, Japan), and energy dispersive X-ray spectroscopy (EDX mapping, H-800, Hitachi, Tokyo, Japan).

Thermogravimetry-differential scanning calorimetric analyses (TG-DSC) were performed using a NETZSCH STA449F3 (Selb, Germany) in the range of 50–800 °C at a heating rate of 10 °C·min^−1^ under flowing argon atmosphere.

The electrochemical properties were tested using CR2032 coin-type test cells (Shenzhen Kejing, Shenzhen, China) with lithium metal foil as anode. The cathode was prepared from a mixture of 60% LiMn*_x_*Fe_1−*x*_PO_4_/C, 10% acetylene black, 20% conductive graphite, and 10% PTFE (polytetrafluoroethylene) in weight. The mixture was cut into rounded slices as a test electrode. The polypropylene film (Celgard 2400, Celgard, NC, USA) was used as separator. Ethylene carbonate/dimethyl carbonate/ethyl methyl carbonate (EC:DMC:EMC = 1:1:1 by volume) solution containing 1 mol·L^−1^ LiPF_6_ was used as the electrolyte. Charge-discharge cycles were carried out on a Land CT2001A battery test system (Shanghai Chenhua Instrument Company, Shanghai, China).

## 3. Results and Discussion

### 3.1. Structures and Morphologies Characterization

**Figure 1:**
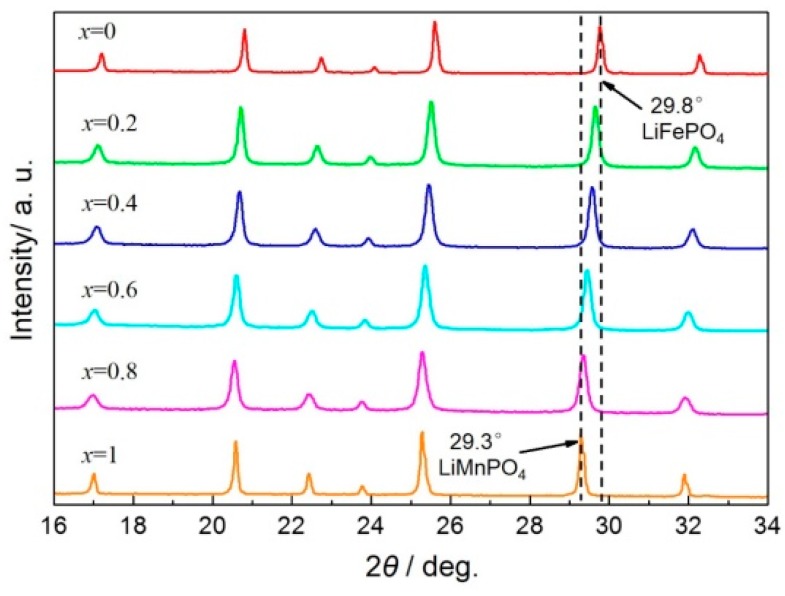
XRD patterns of LiMn*_x_*Fe_1−*x*_PO_4_/C composites.

The XRD patterns of the LiMn*_x_*Fe_1−*x*_PO_4_/C composites with various values of *x* (*x* = 0, 0.2, 0.4, 0.6, 0.8, 1) are shown in Figure 1. An obvious trend of peak shift can be observed in Figure 1 with the increase of *x* from 0 to 1, while the crystalline peaks for LiMn*_x_*Fe_1−*x*_PO_4_ (0 < *x* < 1) all fit with those of LiMnPO_4_ and LiFePO_4_, indicative of pure olivine phase. It is known that a mixture of LiMnPO_4_ and LiFePO_4_ will show double peak 29.3° and 29.8°, which can be indexed to LiMnPO_4_ (PDF 74-0375) and LiFePO_4_ (PDF 81-1173), respectively, so the singe peaks for LiMn*_x_*Fe_1−*x*_PO_4_ (0 < *x* < 1) illustrate completed solid-reaction between LiMnPO_4_ and LiFePO_4_ precursors even under the condition of carbon-coating. Moreover, the shift in the XRD patterns is related with the molar ratio of Mn and Fe in the as-prepared LiMn*_x_*Fe_1−*x*_PO_4_/C composites.

**Figure 2:**
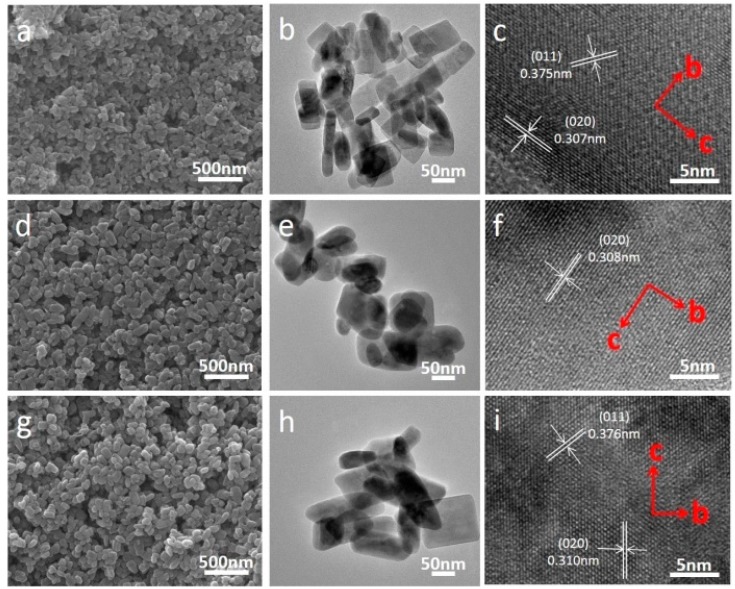
SEM, TEM, and FFT images of LiMn*_x_*Fe_1−*x*_PO_4_: (**a**–**c**) *x* = 1; (**d**–**f**) *x* = 0.4; (**g**–**i**) *x* = 0. LiMn_0.4_Fe_0.6_PO_4_/C inherits plate-like morphology and crystal growth orientation along the *bc* plane of the precursors. No agglomeration and particle size growth are observed.

**Figure 3:**
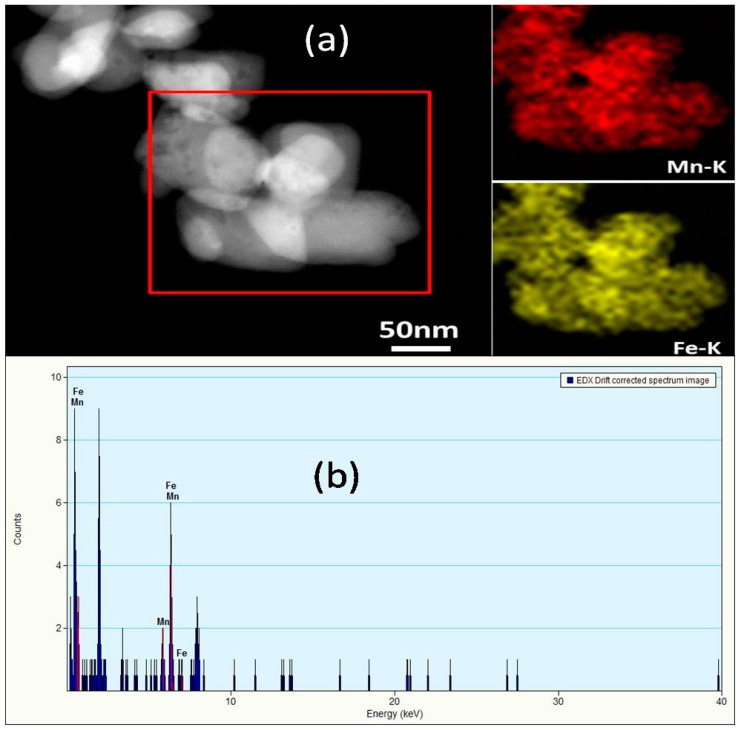
(**a**) Element distribution mappings of Mn and Fe and (**b**) EDX spectrum of LiMn_0.4_Fe_0.6_PO_4_.

Figure 2 shows the SEM and TEM images of the LiMn*_x_*Fe_1−*x*_PO_4_/C composite with *x* = 0, 0.4, 1. It is quite clearly seen that, after heat treatment, the LiMn_0.4_Fe_0.6_PO_4_/C composite still retains similar morphology and size distribution like the precursors. Moreover, the crystal growth orientation of LiMn_0.4_Fe_0.6_PO_4_/C composite is preferable along *bc*-facet, inheriting the orientation of the precursors. The element distribution mappings of Mn and Fe and the EDX spectrum of LiMn_0.4_Fe_0.6_PO_4_ gained from TEM are demonstrated in Figure 3. It can be seen that the distribution of Mn perfectly matches that of Fe, once again certificating a completed solid-reaction between LiMnPO_4_ and LiFePO_4_. The carbon layer on the surface of as-prepared LiMn_0.4_Fe_0.6_PO_4_/C composite is also confirmed by TEM images (Figure S1), which is approximately 2 nm thick.

**Figure 4:**
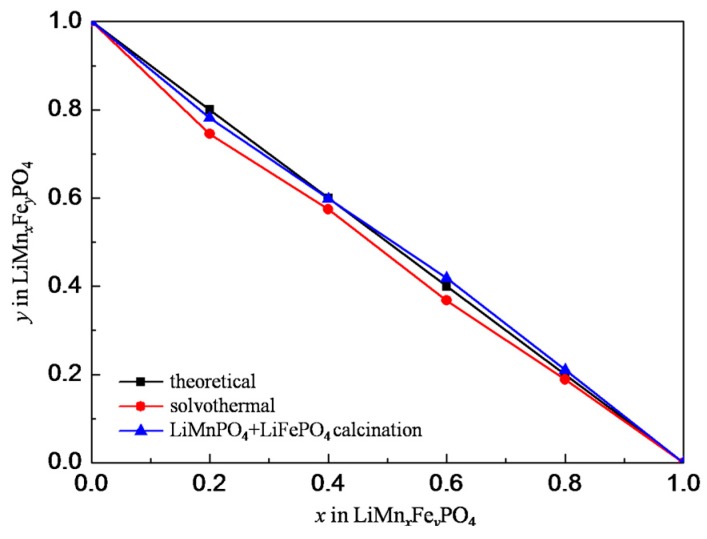
*x*:*y* in LiMn*_x_*Fe*_y_*PO_4_ synthesized via different methods.

To compare Mn–Fe of LiMn*_x_*Fe*_y_*PO_4_ prepared from this novel method and the solvothermal process, ICP-OES analysis was performed and the result is shown in Figure 4. The solvothermal curve lies below the theoretical curve, which means the proportions of Fe in the solvothermal products are less than the theoretical proportion, and the utilization efficiency of Mn is higher than that of Fe during the solvothermal reaction. Different reactivity of Mn^2+^ and Fe^2+^ during the solvothermal reaction may be responsible for this observation. Similar phenomenon for LiMn_0.9_Fe_0.1_PO_4_ has also been reported [8]. The difference in Mn–Fe between feeding and product seems difficult to eliminate. However, the LiMnPO_4_ and LiFePO_4_ calcination curve lies closer to the theoretical curve and fluctuates on both sides. In this sense, solid-state reaction is beneficial to accurately control Mn–Fe in comparison with the solvothermal. This helps adjust Mn–Fe-designed products as well as improve the utilization efficiency of raw materials.

**Figure 5:**
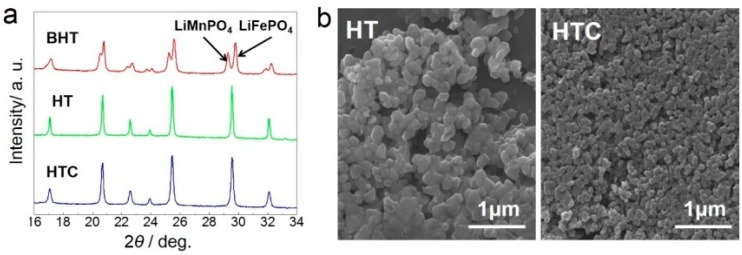
(**a**) XRD Comparison of sample BHT, HT and HTC; (**b**) SEM images of sample HT and HTC. Obvious particle growth and agglomeration can be seen in sample HT.

In conventional solid-state reaction, it is generally difficult to tune the morphology of the product. The morphology and size retention observed in our study can be explained by two reasons. First, LiMnPO_4_ and LiFePO_4_ precursors are plate-like and nano-scaled. Plate-like nanoparticles provide a large contact area and short diffusion path for a solid-state reaction. Second, the carbon from sucrose pyrolysis can prevent further growth of LiMn*_x_*Fe_1−*x*_PO_4_ particles. Figure 5a shows the XRD patterns of BHT (LiMnPO_4_ and LiFePO_4_ precursor mixture before heat treatment), HT (LiMn_0.4_Fe_0.6_PO_4_ without carbon-coating), and HTC (LiMn_0.4_Fe_0.6_PO_4_ with carbon-coating). In particular, the lines lying around 29.5° can provide evidence of mixture or solid solution. BHT presents dual peaks at 29.3° and 29.8°, which can be indexed to LiMnPO_4_ (PDF 75-0375) and LiFePO_4_ (PDF 81-1173), respectively. However, HT and HTC both present a single peak at 29.5°, indicating the solid solution behavior of the as-prepared LiMn_0.4_Fe_0.6_PO_4_. Besides, seen in Table 1, the fwhm (full width at half maximum, criterion of grain size calculation) increase of the HT sample, compared with the HTC sample, proves the inhibition of particle growth when precursors are heated with sucrose. We can also see obvious particle growth and agglomeration of the HT sample in Figure 5b, proving that sucrose has an inhibiting effect on particle growth.

**Figure 6:**
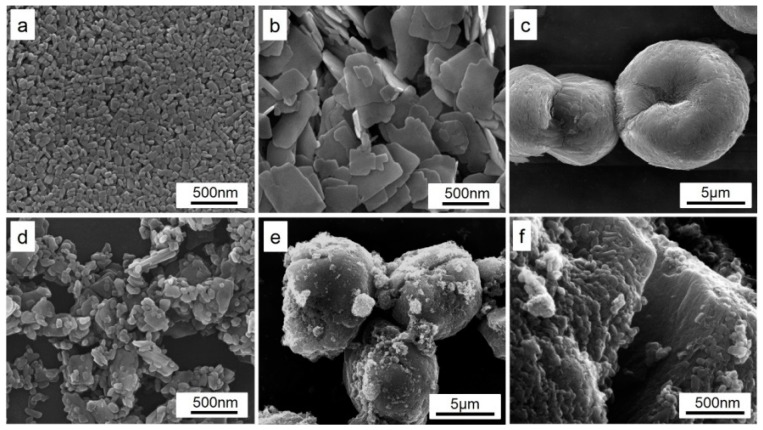
SEM images of LiMnPO_4_ and LiFePO_4_ with different morphology and as-prepared LiMn_0.4_Fe_0.6_PO_4_: (**a**) LiMnPO_4_ nano-plates with lengths less than 100 nm; (**b**) LiFePO_4_ plates with lengths around 500 nm; (**c**) a LiFePO_4_ micro-sphere with a diameter of over 5 μm; (**d**) LiMn_0.4_Fe_0.6_PO_4_ calcined from a + b; (**e**,**f**) LiMn_0.4_Fe_0.6_PO_4_ calcined from a + c.

**Figure 7:**
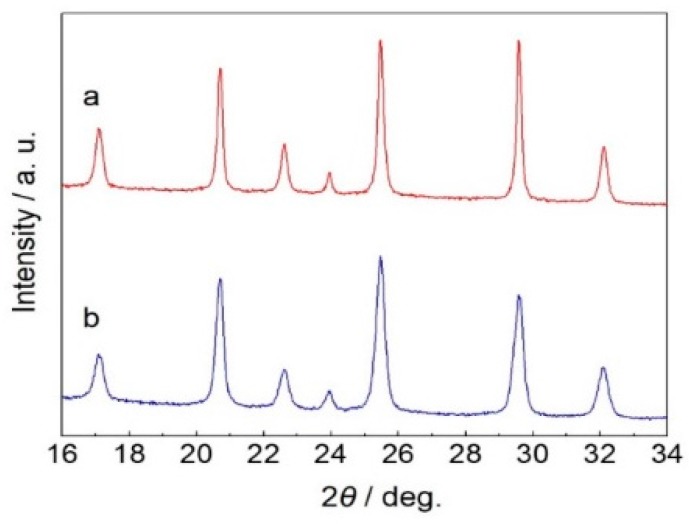
XRD patterns of LiMn_0.4_Fe_0.6_PO_4_ calcined from LiMnPO_4_ and LiFePO_4_ with different morphology: (**a**) LiMnPO_4_ nano-plates with lengths of less than 100 nm + LiFePO_4_ plates with lengths of around 500 nm; (**b**) LiMnPO_4_ nano-plates with lengths of less than 100 nm + a LiFePO_4_ micro-sphere with a diameter of over 5 μm.

To exhibit the inheritance of morphology, precursor plates with different morphologies are mixed and heat-treated to obtain the LiMn_0.4_Fe_0.6_PO_4_/C composite. As seen in Figure 6, (a) LiMnPO_4_ nano-plates with lengths of less than 100 nm were respectively calcined with (b) LiFePO_4_ plates with lengths of around 500 nm or (c) a LiFePO_4_ micro-sphere with a diameter of over 5 μm. In Figure 6d, we can see particles with lengths less than 100 nm as well as around 400–500 nm. Meanwhile, in Figure 6e,f, cracked micro-spheres attached with nanoparticles can be observed. The cracks probably result from the fracture of the micro-sphere when calcined at high temperature. LiMn_0.4_Fe_0.6_PO_4_/C samples with hybrid morphology were obtained, simultaneously inheriting the morphologies of both LiMnPO_4_ and LiFePO_4_ precursors. The XRD patterns presented in Figure 7 indicate a completed solid-state reaction between LiMnPO_4_ and LiFePO_4_ precursors despite their quite different morphologies.

**Figure 8:**
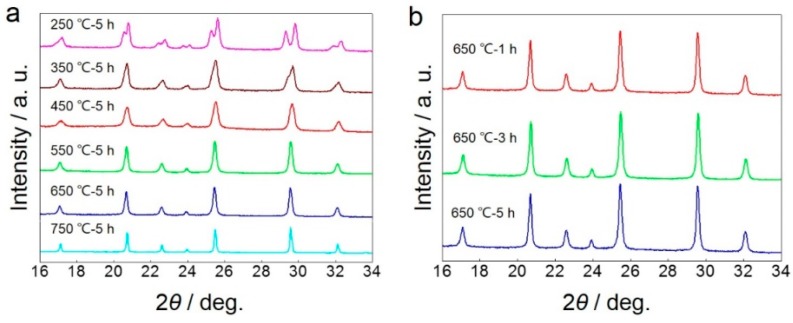
XRD patterns of LiMn_0.4_Fe_0.6_PO_4_ samples synthesized without sucrose (**a**) at various calcination temperature; (**b**) for various time at 650 °C.

The XRD patterns of LiMn_0.4_Fe_0.6_PO_4_ samples synthesized without sucrose at various calcination temperature and time are shown in Figure 8. It can be seen from Figure 8a that, when calcined at 250 °C for 5 h, no visible solid-state reaction occurs between LiMnPO_4_ and LiFePO_4_ for the obvious dual peaks in the XRD pattern. However, the peaks undergo an evolution from dual to unimodal when the calcination temperature rises up to 350 °C. This observation proves that the formation of solid solution can proceed at no more than 350 °C. With calcination temperature sequentially rising up to 450 °C, dual peaks disappear and a solid-state reaction occurs. In addition, the fwhm of XRD patterns decreases as calcination temperature heightens from 450 to 750 °C, indicating that particles grow at high temperatures when calcined without sucrose, consistent with the result shown in Figure 5b. When calcined at 650 °C for a different time, as seen in Figure 8b, a solid-state reaction proceeds fast and occurs within 1 h. Considering the sufficiency of carbon coating, a calcination strategy of relatively higher temperature and longer time (650 °C and 5 h) is necessary.

**Figure 9:**
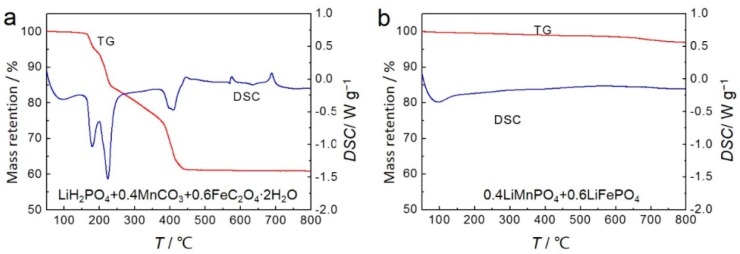
TG-DSC analyses of reaction process of synthesizing LiMn_0.4_Fe_0.6_PO_4_: (**a**) conventional solid-state reaction; (**b**) calcination through precursor nano-plates.

TG-DSC analyses were performed to determine the process of the solid-state reaction. Conventional solid-state synthesis of LiMn_0.4_Fe_0.6_PO_4_ using LiH_2_PO_4_, MnCO_3_, and FeC_2_O_4_·2H_2_O was performed and is shown in Figure 9a. The weight loss started at 160 °C and finished at 450 °C. Three endothermic peaks at 180 °C, 224 °C, and 410 °C were assigned to the evaporation of dehydrated water from FeC_2_O_4_·2H_2_O, the thermal decomposition of MnCO_3_, and the thermal decomposition of FeC_2_O_4_. Additionally, there are two small exothermic peaks at 575 °C and 688 °C without weight loss in the TG profile, which might be attributed to the crystal transform and the lattice heat of LiMn_0.4_Fe_0.6_PO_4_. However, during heat treatment of LiMnPO_4_ and LiFePO_4_ nano-plate mixture, seen in Figure 9b, no peaks are observed in TG-DSC curves above 200 °C. This indicates that Mn^2+^ and Fe^2+^ diffusion between LiMnPO_4_ and LiFePO_4_ phases are dominant during heat treatment since there is no concentration difference of Li^+^ and PO_4_^3−^ between the two phases. Mn^2+^ and Fe^2+^ diffusion can easily proceed through particle boundaries at low temperatures without any exothermic or endothermic processes.

According to the research of Jongsoon Kim [20], the thermal stability of fully delithiated Mn*_x_*Fe_1−*x*_PO_4_ and partially delithiated Li_1−*y*_Mn*_x_*Fe_1−*x*_PO_4_ (0 < *x* < 1, *y* ≈ 0.6) is relatively poor. Mn*_x_*Fe_1−*x*_PO_4_ and Li_1−*y*_Mn*_x_*Fe_1−*x*_PO_4_ would decompose into other phases such as (Mn*_x_*Fe_1−*x*_)_3_(PO_4_)_2_, (Mn*_x_*Fe_1−*x*_)_2_P_2_O_7_, and LiMn*_x_*Fe_1−*x*_PO_4_ at different temperatures according to different *x*-values. The thermal stability of delithiated LiMn*_x_*Fe_1−*x*_PO_4_ is influenced sensitively by the Fe–Mn content in the structure. Nevertheless, with our analysis of the process of the solid-state reaction of theLiMn*_x_*Fe_1−*x*_PO_4_ synthesis, we conclude that fully lithiated LiMn*_x_*Fe_1−*x*_PO_4_ is thermodynamically stable for all *x*-values ranging from 0 to 1 and temperatures ranging from 450 to 800 °C, and the reaction energy barrier to form a LiMn*_x_*Fe_1−*x*_PO_4_ solid solution from LiMnPO_4_ and LiFePO_4_ precursors is quite low. The LiMn*_x_*Fe_1−*x*_PO_4_ phase separation of LiMn*_x_*Fe_1−*x*_PO_4_ can be ignored during preparation. Even if LiMnPO_4_ and/or LiFePO_4_ phases do exist during synthesis, they can react with each other and reunite into a LiMn*_x_*Fe_1−*x*_PO_4_ solid solution during carbon-coating treatment.

### 3.2. Electrochemical Performancesof LiMn_x_Fe_1−x_PO_4_/CMaterials

**Figure 10:**
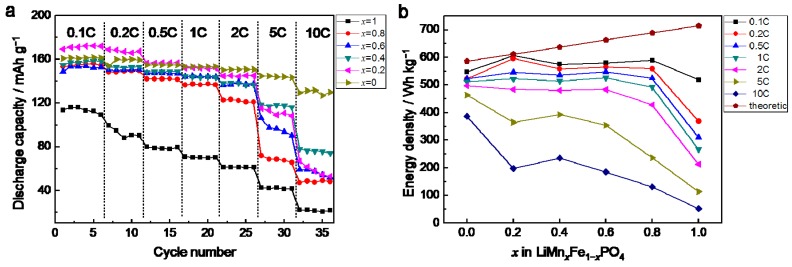
Rate property of LiMn*_x_*Fe_1−*x*_PO_4_/C: (**a**) discharge capacity at different rates; (**b**) variation of energy density at different rates.

The cycling property of LiMPO_4_ (M = Mn, Fe) is recognized as excellent [21,22,23], which can also be confirmed from Figure S2. For LiMn_0.4_Fe_0.6_PO_4_/C composite material prepared by LiMnPO_4_ nano-plates and LiFePO_4_ nano-plates, after 50 cycles at 0.1 C (1 C = 170 mA·g^−1^), the capacity retention is higher than 98%, exhibiting excellent cycling stability. The half-cells are charged at 0.1 C and discharged at various C-rates to help sufficient delithiation and to remove the side effect of discharging. As shown in Figure 10a, discharge capacity at 5 C is 42.8 mAh·g^−1^, 72.1 mAh·g^−1^, 106.3 mAh·g^−1^, 118.9 mAh·g^−1^, 114.9 mAh·g^−1^, and 144.9 mAh·g^−1^, corresponding to *x* = 0, 0.2, 0.4, 0.6, 0.8, and 1, respectively. LiMn_0.4_Fe_0.6_PO_4_/C shows an outstanding high rate property of 78 mAh·g^−1^ at 10 C, reaching up to 50% retention of that at 0.1 C, comparable to other previously reported research [14,24,25]. Figure 10b shows the variation of energy density performing at different discharge rates. At a low discharging rate (0.1 to 0.2 C), LiMn_0.2_Fe_0.8_PO_4_/C shows outstanding high energy density nearly 600 Wh·kg^−1^, reaching very close to theoretic value of 612 Wh·kg^−1^. With discharging rate getting higher (0.5 to 2 C), energy density of LiMn*_x_*Fe_1−*x*_PO_4_/C remains nearly unchanged when *x* lands in the range of 0 to 0.8, promoting LiMn*_x_*Fe_1−*x*_PO_4_/C to a role of tolerant material for stable energy storage at low current density. The energy density of LiMn_0.4_Fe_0.6_PO_4_/C at 5 C and 10 C is 393.2 Wh·kg^−1^ and 235.6 Wh·kg^−1^, which makes LiMn_0.4_Fe_0.6_PO_4_/C a promising material for high-energy applications.

## 4. Conclusions

LiMn*_x_*Fe_1−*x*_PO_4_/C nano-plates with regulated morphology and accurate stoichiometry are synthesized through a novel solid-state reaction of solvothermal-prepared LiMnPO_4_ and LiFePO4 nano-plates under the condition of carbon coating with sucrose. For the benefit of carbonated sucrose, a LiMn*_x_*Fe_1−*x*_PO_4_/C composite inherits the morphology, crystalline structure, and particle size of LiMnPO_4_ and LiFePO_4_ precursors. Ion diffusion of Mn^2+^ and Fe^2+^ can proceed easily through LiMnPO_4_ and LiFePO_4_ phases at only around 350 °C to form a thermodynamic stable LiMn*_x_*Fe_1−*x*_PO_4_ phase. With the optimization of *x* in LiMn*_x_*Fe_1−*x*_PO_4_/C, the LiMn_0.4_Fe_0.6_PO_4_/C composite shows excellent high rate discharge capacity of 118.9 mAh·g^−1^ at 5 C and 78 mAh·g^−1^ at 10 C, equivalent to 393.2 Wh·kg^−1^ and 235.6 Wh·kg^−1^ in terms of energy density. This paves a novel and facile way to synthesize LiMn*_x_*Fe_1−*x*_PO_4_ material with low cost, high energy density, and stability for lithium ion batteries.

## Figures and Tables

**Table 1:** fwhm of HT and HTC at different peaks.

Peak Position/°	17.1	20.7	22.6	23.9	25.5	29.5	32.1
fwhm of HT/°	0.138	0.139	0.143	0.140	0.149	0.132	0.149
fwhm of HTC/°	0.216	0.182	0.241	0.187	0.209	0.193	0.233

## Supplementary Materials

The following are available online at www.mdpi.com/1996-1944/9/9/766/s1. Figure S1: (a) TEM and (b) magnified TEM images of LiMn_0.4_Fe_0.6_PO_4_/C composite materials; Figure S2: (a) Cycling performance at 0.1 C and (b) voltage profile of LiMn_0.4_Fe_0.6_PO_4_/C composite material prepared by LiMnPO_4_ nano-plates and LiFePO_4_ nano-plates.
