# Supplementary material for: The Synthesis of LiMnxFe1−xPO4/C Cathode Material through Solvothermal Jointed with Solid-State Reaction
Source: Materials (Basel). 2016 Sep 8;9(9):766. doi: 10.3390/ma9090766 (PMC5457063; doi:10.3390/ma9090766)
Supplement: Supplementary file 1 [file materials-09-00766-s001.pdf]

Supplementary Materials: The Synthesis of $LiMn_xFe_{1-x}PO_4/C$ Cathode Material through Solvothermal Jointed with Solid-State Reaction

Xiangming He, Jixian Wang, Zhongjia Dai, Li Wang and Guangyu Tian

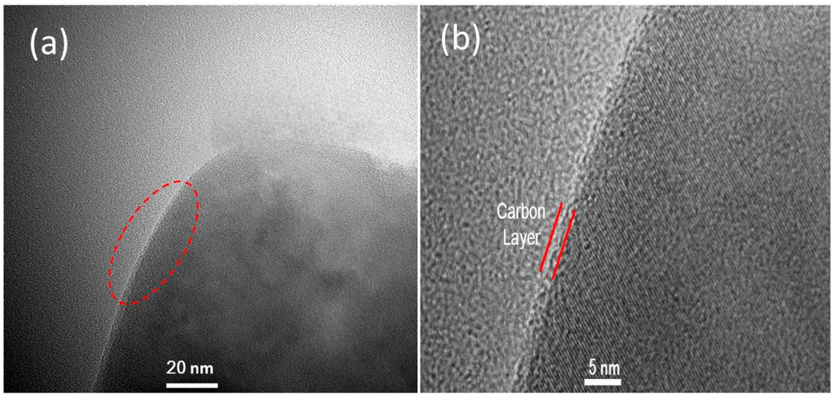

Figure S1. (**a**) TEM and (**b**) magnified TEM images of $LiMn_{0.4}Fe_{0.6}PO_4/C$ composite materials.

Figure S1 shows the TEM and magnified TEM images of the $LiMn_{0.4}Fe_{0.6}PO_4/C$ composite, which are used to verify the carbon layer on the surface of the final product. The images illustrate that there is a carbon layer with a thickness of about 2 nm on the surface of final product after carbon coating from sucrose pyrolysis.

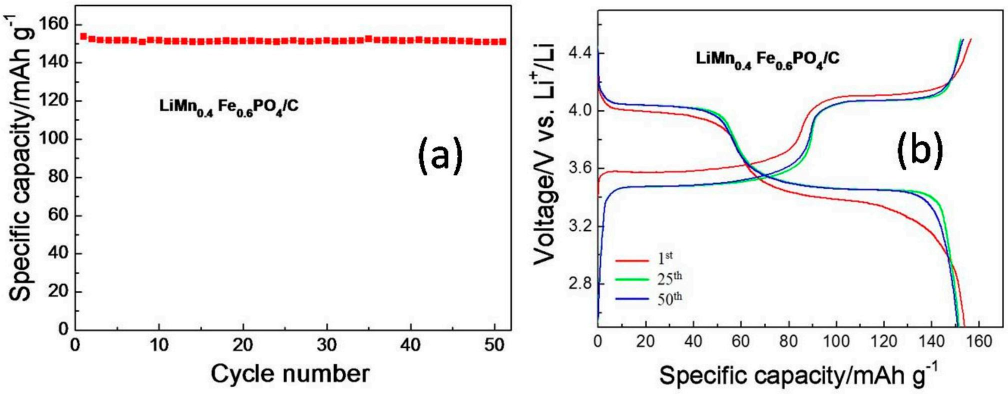

Figure S2. (**a**) Cycling performance at 0.1 C (1 C = 170 mA·g^{-1}) and (**b**) voltage profile of $LiMn_{0.4}Fe_{0.6}PO_4/C$ composite material prepared by $LiMnPO_4$ nano-plates and $LiFePO_4$ nano-plates.

Figure S2 shows the cycling performance and voltage profile of the $LiMn_{0.4}Fe_{0.6}PO_4/C$ composite materials. After 50 cycles at 0.1 C, the capacity retention is higher than 98%. It can be clearly seen that $LiMn_{0.4}Fe_{0.6}PO_4/C$ composite material exhibits excellent cycling stability.
